# Examining the continuum of resistance model in two population-based screening studies in Sweden

**DOI:** 10.1016/j.pmedr.2023.102317

**Published:** 2023-07-13

**Authors:** Anton Nilsson, Ulf Strömberg, Jonas Björk, Anna Forsberg, Kaisa Fritzell, Katrin Ragna Kemp Gudmundsdottir, Johan Engdahl, Carl Bonander

**Affiliations:** a Epidemiology, Population Studies and Infrastructures (EPI@LUND), Lund University, Lund, Sweden; b Region Halland, Halmstad, Sweden; c Health Economics and Policy, School of Public Health & Community Medicine, Institute of Medicine, University of Gothenburg, Gothenburg, Sweden; d Clinical Studies Sweden, Forum South, Skåne University Hospital, Lund, Sweden; e Department of Medicine, Solna, Karolinska Institutet, Stockholm, Sweden; f Department of Neurobiology, Care Sciences and Society, Division of Nursing, Karolinska Institutet, Stockholm, Sweden; g The Hereditary Cancer Clinic, Theme Cancer, Karolinska University Hospital, Stockholm, Sweden; h Department of Clinical Sciences, Danderyd Hospital, Karolinska Institutet, Stockholm, Sweden; i Centre for Societal Risk Research, Karlstad University, Sweden

**Keywords:** Generalizability, Selection, Screening, Atrial fibrillation, Colorectal cancer, Continuum of resistance

## Abstract

In studies recruited on a voluntary basis, lack of representativity may impair the ability to generalize findings to the target population. Previous studies, primarily based on surveys, have suggested that generalizability may be improved by exploiting data on individuals who agreed to participate only after receiving one or several reminders, as such individuals may be more similar to non-participants than what early participants are. Assessing this idea in the context of screenings, we compared sociodemographic characteristics and health across early, late, and non-participants in two large population-based screening studies in Sweden: STROKESTOP II (screening for atrial fibrillation; 6,867 participants) and SCREESCO (screening for colorectal cancer; 39,363 participants). We also explored the opportunities to reproduce the distributions of characteristics in the full invited populations, either by assuming that the non-participants were similar to the late participants, or by applying a linear extrapolation model based on both early and late participants. Findings showed that early and late participants exhibited similar characteristics along most dimensions, including civil status, education, income, and health examination results. Both these types of participants in turn differed from the non-participants, with fewer married, lower educational attainments, and lower incomes. Compared to early participants, late participants were more likely to be born outside of Sweden and to have comorbidities, with non-participants similar or even more so. The two empirical models improved representativity in some cases, but not always. Overall, we found mixed support that data on late participation may be useful for improving representativeness of screening studies.

## 1 Introduction

Lack of representativeness is a concern in studies recruited on a voluntary basis. Indeed, evidence from different contexts, including health surveys (Cunradi et al., 2005, Eagan et al., 2002, Galea and Tracy, 2007, Hill et al., 1997, Nilsson et al., 2021b, Van Loon, 2003) and screenings (Engdahl et al., 2016, Linne et al., 2014, Maheswaran et al., 2006, McLachlan et al., 2012, Partin et al., 2003, Zarrouk et al., 2013) has shown that certain groups, such as those with low socioeconomic status, immigrants, unmarried people, and smokers, are less likely to participate. Participation in screening has additionally been found to be related to factors such as proximity to the screening site, perceived benefit of the screening, and feelings of vulnerability (Engdahl et al., 2016, Linne et al., 2014, McLachlan et al., 2012, McQueen et al., 2010). If these or other factors related to study participation are also associated with disease prevalence or incidence, benefits of screening, or other outcomes under study, study results will fail to generalize, at least without proper adjustments (Lesko et al., 2017, Nilsson et al., 2021a).

There is growing interest in examining lack of representativeness in epidemiologic studies (Batty et al., 2020, Bonander et al., 2019, Ferrie et al., 2009, Hara et al., 2002, Mattila et al., 2007, Nilsen et al., 2009) and in developing and applying models to try to correct for it (Bonander et al., 2019, Buchanan et al., 2018, Lesko et al., 2017, Nilsson et al., 2021b, Nilsson et al., 2020, Pearl, 2015). The *continuum of resistance model* (Filion, 1976, Lin and Schaeffer, 1995) may be useful in settings where recruitment involves one or several reminders, and where there are data on whether participants agreed to participate already after the first invitation or only after one or several reminders. According to the model, individual characteristics follow a gradient with respect to the individual’s propensity to participate in a study. Hence, for any characteristic whose average or proportion differs between individuals with a high and low participation propensity, the average or proportion of the same characteristic among individuals with an intermediate participation propensity should be found somewhere in between those among individuals high and low participation propensities. In addition, the model assumes that the propensity to participate in a study can be measured by whether an individual agreed to participate in the study without reminders (“early participation”), after one or several reminders (“late participation”), or if they refused to participate altogether (non-participation). Given these assumptions, data on early and late participation can potentially be used to estimate results for the entire invited population, either via an extrapolation model (Filion, 1976, Filion, 1975) or by (conservatively) assuming that non-participants are similar to those who agree to participate only after reminders (Boniface et al., 2017, Kypri et al., 2011, Maclennan et al., 2012).

Evidence on the continuum of resistance model comes mostly from studies based on surveys. Several of these have provided evidence in favor of the model, insofar as that late responders differed from early responders in ways that were at least qualitatively resemblant of how responders often differ from non-responders; for example, having lower socioeconomic status and more adverse health-related behaviors (Boniface et al., 2017, Clarsen et al., 2021, Klingwort et al., 2018, Kypri et al., 2011, Maclennan et al., 2012, Studer et al., 2013). Some studies had access to data on certain characteristics also among non-responders and could confirm that late responders resembled non-responders more than what early responders did (Maclennan et al., 2012, Studer et al., 2013). In other studies, however, little, no, or mixed evidence of a continuum was found (Etter et al., 1997, Lahaut et al., 2003, Paganini-Hill et al., 1993, Zhao et al., 2009).

To date, there is little evidence on the continuum of resistance model in the context of screenings or health examinations. However, as the determinants of participation in screenings and health examinations differ from those in surveys, it is unclear to what extent findings from the latter context would extend to the former. A German study based on a combination of self-reports and examinations of common chronic conditions such as hypertension and diabetes found little evidence of a continuum of resistance (Haring et al., 2009). An Australian study of screening for colorectal cancer found mixed evidence (Gregory et al., 2013), but was limited by a small sample.

STROKESTOP II and SCREESCO are two large Swedish population-based screening studies; STROKESTOP II is a study of screening for atrial fibrillation (AF), and SCREESCO is a study of screening for colorectal cancer. In the present work, we assessed the applicability of the continuum of resistance model by comparing early, late, and non-participants in these studies with respect to a range of background characteristics from national registers. The potential to reproduce the distributions of characteristics in the full invited populations without utilizing information on non-participants was explored both with a model assuming that non-participants were similar to late participants and an extrapolation approach based on contrasts between early and late participants. By examining the ability of these methods to reproduce distributions of characteristics observable in the full invited populations, we provided indications of whether these may also be able to reproduce distributions of outcomes not observable in the full invited populations, such as prevalence of risk factors screened for, and long-term benefits of screening.

## 2 Materials and methods

### 2.1 Study populations and data

#### 2.1.1 STROKESTOP II

The study base in STROKESTOP II consisted of all individuals born in 1940 or 1941, living in Stockholm County by the end of 2015 (n = 28,712) (Engdahl et al., 2017, Gudmundsdottir et al., 2022, Gudmundsdottir et al., 2021, Gudmundsdottir et al., 2020). Half of this population were randomized to be invited to screening for AF. As some individuals had died, emigrated or could not be reached, 14,231 were sent invitations. Initial invitations were sent out between April 2016 and September 2017. Individuals not participating after the first invitation were sent up to two reminders; the first round of reminders was sent out between December 2016 and November 2017 and the second between May 2017 and January 2018. Invited individuals who agreed to participate and who had no history of AF were screened for this outcome through a combination of ECG and serum measurements of N-terminal pro b-type natriuretic peptide (NT-proBNP).

We exploited data on whether STROKESTOP II participants agreed to participate already after the first invitation or only after one or two reminders. Moreover, we obtained data from administrative registers held by Statistics Sweden (SCB) and the National Board of Health and Welfare. These data covered the full population of STROKESTOP II invitees and were linked to the participant data using personal identifiers. Data from SCB encompassed sociodemographics including sex, education, income (individual disposable income of less than 150,000 SEK/year, between 150,000 and 300,000 SEK/year, or more than 300,000 SEK/year), immigrant status (immigrant or born in Sweden), and civil status by the end of 2015. There was also information on death and migration dates. Data from the National Board of Health and Welfare included inpatient and outpatient hospital visits, spanning 2001–2015. For participants, we also used data on blood pressure, NT-proBNP, and AF, as determined at the screenings.

#### 2.1.2 SCREESCO

The SCREESCO study was based on individuals aged 60, living in 18 out of Sweden’s 21 regions (Forsberg et al., 2022, Strömberg et al., 2022). Individuals from this population were drawn randomly and allocated to one of two intervention arms: SCREESCO-FIT (n = 60,300), where individuals were invited to return two kits with stool samples (analyzed with a fecal immunochemical test; FIT), and SCREESCO-COL (n = 31,140), where individuals were invited to undergo colonoscopy. Initial invitations to SCREESCO-FIT were sent out between April 2014 and March 2017, whereas initial invitations to SCREESCO-COL were sent out between March 2014 and December 2019. In both arms, a reminder was sent out eight weeks after the initial invitation unless the individual had responded to the initial one. Individuals with a positive test result in SCREESCO-FIT were additionally referred to colonoscopy. Moreover, all participants in SCREESCO-FIT were invited to return two additional kits with stool samples two years after their first invitation.

In the present work, we considered both arms of SCREESCO. For simplicity, however, we only included the initial home tests in the FIT arm, thus disregarding the potential colonoscopies and two-year follow-up tests also conducted within this arm. After removal of individuals who had died, emigrated from Sweden, or for other reasons could not be reached, there were 60,137 invited individuals in the FIT arm and 30,400 in the COL arm.

Besides data on which study arm individuals were assigned to, whether they agreed to participate, and if so with or without a reminder, we again had access to sociodemographics from SCB, linked by personal identifiers. These included sex, education, income (four quartiles of household disposable (Strömberg et al., 2022)), immigrant status, and civil status, all measured by the end of the year before the invitation. For participants in SCREESCO-FIT, we also used data on whether their home test yielded a positive result. Data on colonoscopy yield was not available.

### 2.2 Statistical analyses

In STROKESTOP II, we compared four groups of individuals: 1) those who agreed to participate already after one invitation, 2) those who agreed to participate after one reminder, 3) those who agreed to participate after two reminders, and 4) those who were invited but did not participate. These groups were compared with respect to sociodemographics and the Charlson comorbidity index (Charlson et al., 1987). We also compared blood pressure, NT-proBNP, and the prevalence of detected AF across groups 1–3. Furthermore, in each arm of SCREESCO, we compared sociodemographics across groups 1–2 and 4, defined similarly as above, and compared the prevalence of a positive test result from the home test across groups (1), (2). Throughout, confidence intervals were obtained with normal approximation.

We then examined the opportunities to approximate characteristics in the full invited populations without using data on non-participants. As a first approach (Boniface et al., 2017, Kypri et al., 2011, Maclennan et al., 2012) – which we refer to as “the substitution method” – we exploited that a population average or proportion can be written as a weighted average of its group-specific components:

(1) $$\overset{¯}{y_{\mathrm{pop}}}=\alpha \overset{¯}{y_{\alpha }}+\beta \overset{¯}{y_{\beta }}+\gamma \overset{¯}{y_{\gamma }}+(1-\alpha -\beta -\gamma )\overset{¯}{y_{n}}$$

**Fig. 1:**
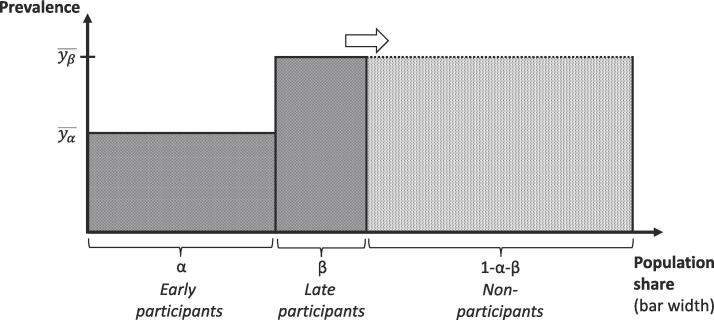
The substitution method (an example with only one group of late participants) Early participants make up the share α of the full population, late participants β, and non-participants 1-α-β. The proportion with the characteristic of interest is $\overset{¯}{y_{\alpha }}$ among early participants and $\overset{¯}{y_{\beta }}$ among late participants. The model assumes that non-participants are similar to late participants on average. The estimated population proportion $\overset{¯}{y_{\mathrm{pop}}}$ is given by $\alpha \overset{¯}{y_{\alpha }}+(1-\alpha )\overset{¯}{y_{\beta }}$, which lies somewhere in between $\overset{¯}{y_{\alpha }}$ and $\overset{¯}{y_{\beta }}$ and corresponds to the combined area of the dark and light shaded regions.

Here, α, β, and γ are the population shares of groups 1–3 previously defined (γ=0 in SCREESCO); $\overset{¯}{y_{\alpha }}$, $\overset{¯}{y_{\beta }}$, $\overset{¯}{y_{\gamma }}$, and $\overset{¯}{y_{n}}$ are the four group-specific averages or proportions of the variable of interest. To implement the substitution method, we set $\overset{¯}{y_{n}}$ equal to the corresponding value in the group least prone to participate ($\overset{¯}{y_{\beta }}$ in SCREESCO and $\overset{¯}{y_{\gamma }}$ in STROKESTOP II), and then calculated $\overset{¯}{y_{\mathrm{pop}}}$ according to the equation. Normal-based confidence intervals were obtained. The approach is illustrated in Fig. 1.

As an alternative approach – “the extrapolation method” – we estimated the following linear model (Filion, 1976, Filion, 1975):

(2) $$\overset{¯}{y}=a+b\overset{¯}{x}+e$$

**Fig. 2:**
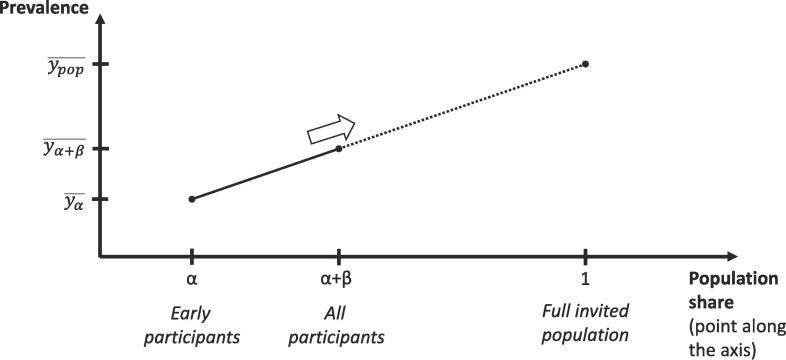
The extrapolation method (an example with only one group of late participants) Early participants make up the share α of the full population and late participants β, implying that all participants combined make up α+β. The proportion with the characteristic of interest is $\overset{¯}{y_{\alpha }}$ among early participants and $\overset{¯}{y_{\alpha +\beta }}$ among all participants combined. The model assumes that differences between early participants and the full participant group are informative about differences between the full participant group and the full invited population, according to a linear model. The predicted population proportion is given by $\overset{¯}{y_{\mathrm{pop}}}$.

Here, $\overset{¯}{y}$ represents a *cumulative average or proportion* of some characteristic, whereas $\overset{¯}{x}$ represents a *cumulative population share*. For each outcome, the equation was estimated with ordinary least squares, based on two or three pairs of $\overset{¯}{y}$ and $\overset{¯}{x}$. The first pair represented participants who agreed to participate already after the first invitation, with $\overset{¯}{y}$ measuring their average characteristic and $\overset{¯}{x}$ being their share of the population. The second pair represented the corresponding values if considering participants who either agreed to participate after the first invitation or after one reminder. In STROKESTOP II, a third pair was formed based on those who agreed to participate either after the first invitation or after one or two reminders. Having estimated the parameters in the equation, we set $\overset{¯}{x}$ equal to 1 to predict $\overset{¯}{y}$ for a hypothetical cumulative participation proportion of 1, that is, for the full invited population. Normal-based confidence intervals were obtained by bootstrapping the entire procedure. No covariates other than $\overset{¯}{x}$ were included. The approach is illustrated in Fig. 2.

Data preparation was done in R version 4.1.2 and statistical analyses in Stata version 16.1 (StataCorp). The Charlson comorbidity index was calculated based on inpatient and outpatient hospital visits using the *comorbidity* package (version 1.0.2; (Gasparini, 2018)) in R.

### 2.3 Ethical approval

The Stockholm Regional Ethics Committee approved the analysis of STROKESTOP II (2015/2079–31/1) and SCREESCO (2012/2058–31/3). The study was performed in accordance with the Declaration of Helsinki.

## 3 Results

### 3.1 Sample constructions

Omitting STROKESTOP II invitees who were absent in the population registers by 2015 (n = 2), had died (n = 161) or emigrated (n = 4) after 2015 but before receiving their first invitation, 14,064 individuals remained in the invited sample. Of these, 5,495 (39%) agreed to participate after the initial invitation, 970 (7%) after one reminder, 402 (3%) after two reminders, and the remaining 7,197 (51%) did not participate. In SCREESCO, we excluded invitees who were absent in the population registers in the year before their invitation (n = 37 in the FIT arm; n = 5 in the COL arm), yielding 60,100 individuals in the FIT arm and 30,395 in the COL arm. Among those in the FIT arm, 23,036 (38%) agreed to participate after the initial invitation, 5,786 (10%) after a reminder, and the remaining 31,278 (52%) did not participate. Among individuals in the COL arm, 8,131 (27%) agreed to participate after the initial invitation, 2,410 (8%) after a reminder, and the remaining 19,854 (65%) did not participate.

### 3.2 Early, late, and non-participants in STROKESTOP II

Non-participants in STROKESTOP II were more likely than early participants to be male, non-married, lower educated, lower income, and immigrants, and were less healthy according to the Charlson comorbidity index (Table 1). Late participants were similar to early participants with respect to education, income, and civil status. In contrast, late participants were more similar to non-participants than early participants with respect to sex. For immigrant status and comorbidities, the characteristics of late participants were in between those of early and non-participants. Early and late participants were similar with respect to blood pressure, NT-proBNP, and detected AF. Values in the full invited population tended to fall somewhere in between those of late participants and non-participants.

**Table 1:** Averages and proportions (95% confidence intervals) of different characteristics across the four groups considered in STROKESTOP II.

Variable	**Early participants** **(n = 5,495)**	**One reminder** **(n = 970)**	**Two reminders** **(n = 402)**	**Non-participants** **(n = 7,197)**	**True values in full invited population (n = 14,064)**
*Sex*					
Male	0.45 (0.44–0.47)	0.48 (0.43–0.52)	0.50 (0.45–0.54)	0.48 (0.47–0.49)	**0.47** **(0.46**–**0.48)**
*Civil status*					
Married	0.59 (0.57–0.60)	0.60 (0.56–0.63)	0.58 (0.53–0.63)	0.49 (0.48–0.51)	**0.54** **(0.53**–**0.55)**
Unmarried	0.079 (0.072–0.086)	0.089 (0.071–0.11)	0.082 (0.055–0.11)	0.11 (0.10–0.12)	**0.10** **(0.09**–**0.10)**
Divorced	0.21 (0.20–0.22)	0.20 (0.17–0.22)	0.22 (0.18–0.26)	0.25 (0.24–0.26)	**0.23** **(0.22**–**0.23)**
Widowed	0.13 (0.12–0.14)	0.12 (0.10–0.14)	0.12 (0.09–0.15)	0.15 (0.14–0.16)	**0.14** **(0.13**–**0.14)**
*Education*					
Primary	0.17 (0.16–0.18)	0.20 (0.18–0.23)	0.19 (0.15–0.22)	0.29 (0.28–0.30)	**0.24** **(0.23**–**0.24)**
Secondary	0.41 (0.40–0.42)	0.39 (0.36–0.42)	0.37 (0.32–0.42)	0.40 (0.39–0.41)	**0.40** **(0.39**–**0.41)**
Tertiary	0.41 (0.40–0.42)	0.38 (0.35–0.42)	0.43 (0.38–0.48)	0.27 (0.26–0.28)	**0.34** **(0.33**–**0.34)**
Missing	0.0067 (0.0046–0.0089)	0.020 (0.011–0.028)	0.017 (0.046–0.030)	0.041 (0.037–0.046)	**0.026** **(0.023**–**0.028)**
*Income*					
Low	0.23 (0.22–0.24)	0.26 (0.24–0.29)	0.28 (0.24–0.33)	0.41 (0.40–0.42)	**0.32** **(0.32**–**0.33)**
Medium	0.53 (0.52–0.54)	0.50 (0.47–0.53)	0.46 (0.41–0.51)	0.45 (0.44–0.46)	**0.49** **(0.48**–**0.49)**
High	0.24 (0.23–0.25)	0.24 (0.21–0.26)	0.26 (0.22–0.30)	0.14 (0.14–0.15)	**0.19** **(0.18**–**0.20)**
*Origin*					
Immigrant	0.16 (0.15–0.17)	0.19 (0.16–0.21)	0.22 (0.18–0.26)	0.27 (0.26–0.28)	**0.22** **(0.21**–**0.23)**
*Morbidity*					
Charlson	0.55 (0.52–0.57)	0.61 (0.55–0.68)	0.62 (0.53–0.72)	0.82 (0.79–0.84)	**0.69** **(0.68**–**0.71)**
Systolic BP	139 (139–140)	139 (138–140)	140 (138–142)	–	–
Diastolic BP	81 (81–82)	81 (80–82)	82 (81–83)	–	–
Log NT-proBNP	5.07 (5.05–5.09)	5.09 (5.04–5.14)	5.17 (5.08–5.25)	–	–
Detected AF	0.025 (0.021–0.029)	0.018 (0.0093–0.026)	0.022 (0.079–0.037)	–	–

Sociodemographic variables were obtained from Statistics Sweden whereas hospitalizations (used to calculate the Charlson comorbidity index) were obtained from the National Board of Health and Welfare. Sociodemographic variables refer to 2015; hospitalizations between 2001 and 2015 were used. Blood pressure (BP), NT-proBNP, and atrial fibrillation (AF) were determined at the screening.

### 3.3 Substitution and extrapolation in STROKESTOP II

In STROKESTOP II, the substitution method provided better approximations for the population averages of immigrant status and comorbidity (Table 2) than the numbers provided by the unadjusted participant group. The method was not, however, superior to this baseline in approximating the distributions of sex, civil status, education, or income.

**Table 2:** Averages and proportions (95% confidence intervals) of different characteristics across invited and participants, and predicted values for the invited population based on early and late participants in STROKESTOP II.

Variable	**True values in full invited population (n = 14,064)**	**Values among participants (n = 6,867)**	**Substitution based on late participants**	**Linear extrapolation**
*Sex*				
Male	**0.47** **(0.46**–**0.48)**	0.46 (0.45–0.47)	0.48 (0.44–0.51)	0.50 (0.46–0.53)
*Civil status*				
Married	**0.54** **(0.53**–**0.55)**	0.59 (0.58–0.60)	0.58 (0.54–0.62)	0.59 (0.56–0.63)
Unmarried	**0.10** **(0.09**–**0.10)**	0.081 (0.074–0.087)	0.081 (0.066–0.097)	0.089 (0.071–0.108)
Divorced	**0.23** **(0.22**–**0.23)**	0.21 (0.20–0.22)	0.21 (0.19–0.24)	0.20 (0.18–0.23)
Widowed	**0.14** **(0.13**–**0.14)**	0.13 (0.12–0.14)	0.12 (0.10–0.14)	0.11 (0.09–0.14)
*Education*				
Primary	**0.24** **(0.23**–**0.24)**	0.18 (0.17–0.19)	0.18 (0.16–0.21)	0.21 (0.18–0.23)
Secondary	**0.40** **(0.39**–**0.41)**	0.41 (0.40–0.42)	0.42 (0.38–0.45)	0.38 (0.35–0.42)
Tertiary	**0.34** **(0.33**–**0.34)**	0.40 (0.39–0.42)	0.39 (0.35–0.42)	0.39 (0.36–0.42)
Missing	**0.026** **(0.023**–**0.028)**	0.0092 (0.0069–0.011)	0.013 (0.0063–0.020)	0.022 (0.013–0.032)
*Income*				
Low	**0.32** **(0.32**–**0.33)**	0.24 (0.23–0.25)	0.26 (0.23–0.29)	0.28 (0.25–0.31)
Medium	**0.49** **(0.48**–**0.49)**	0.52 (0.51–0.53)	0.49 (0.44–0.53)	0.48 (0.45–0.52)
High	**0.19** **(0.18**–**0.20)**	0.24 (0.23–0.25)	0.25 (0.22–0.28)	0.24 (0.21–0.27)
*Origin*				
Immigrant	**0.22** **(0.21**–**0.23)**	0.16 (0.16–0.17)	0.19 (0.17–0.22)	0.21 (0.18–0.23)
*Morbidity*				
Charlson	**0.69** **(0.68**–**0.71)**	0.56 (0.54–0.59)	0.59 (0.54–0.65)	0.63 (0.58–0.69)
Systolic BP	**–**	139 (139–139)	140 (132–147)	139 (138–141)
Diastolic BP	**–**	81 (81–81)	82 (77–86)	81 (80–82)
Log NT-proBNP	**–**	5.08 (5.06–5.10)	5.13 (4.85–5.40)	5.12 (5.06–5.17)
Detected AF	**–**	0.024 (0.020–0.028)	0.023 (0.015–0.031)	0.017 (0.0080–0.026)

Sociodemographic variables were obtained from Statistics Sweden whereas hospitalizations (used to calculate the Charlson comorbidity index) were obtained from the National Board of Health and Welfare. Sociodemographic variables refer to 2015; hospitalizations between 2001 and 2015 were used. Blood pressure (BP), NT-proBNP, and atrial fibrillation (AF) were determined at the screening.

Likewise, the extrapolation method did not improve estimates of the population distributions of sex or civil status, as compared to the baseline of unadjusted STROKESTOP II participants. Estimates for education and income tended to improve, however, and those for immigrant status and comorbidities even more so.

Neither substitution nor extrapolation made much difference to the estimates of mean blood pressure, mean (log) NT-proBNP, or probability of detected AF, as compared to the baseline.

### 3.4 Early, late, and non-participants in SCREESCO

In both arms of SCREESCO, non-participants were more likely than early and late participants to be non-married, lower educated, lower income, and immigrants (Table 3). Patterns for sex were mixed, with non-participants in SCREESCO-FIT being more likely to be male and non-participants in SCREESCO-COL being more likely to be female. Late participants were similar to early participants with respect to civil status, education, and income, but more similar to non-participants with respect to immigrant status. In SCREESCO-FIT, the sex distribution among late participants was similar to that among non-participants, whereas in SCREESCO-COL the share of males was much higher in the late participant group than among both early and non-participants. The probability of a positive FIT result was somewhat higher among late than early participants. In SCREESCO-FIT, values in the full invited cohort tended to be similar to those among late participants, whereas in SCREESCO-COL, values in the full invited cohort tended to be more similar to those among the non-participants, consistent with the lower participation rate.

**Table 3:** Proportions (95% confidence intervals) of different characteristics across the groups considered in SCREESCO.

	**Home test arm (SCREESCO-FIT)**				**Colonoscopy arm (SCREESCO-COL)**			
Variable	**Early participants** **(n = 23,036)**	**Reminded** **(n = 5,786)**	**Non-participants** **(n = 31,278)**	**True values in full invited population (n = 60,100)**	**Early participants** **(n = 8,131)**	**Reminded** **(n = 2,410)**	**Non-participants** **(n = 19,854)**	**True values in full invited population (n = 30,395)**
*Sex*								
Male	0.44 (0.43–0.44)	0.53 (0.52–0.54)	0.54 (0.53–0.54)	**0.50 (0.49**–**0.50)**	0.50 (0.49–0.51)	0.55 (0.53–0.58)	0.49 (0.48–0.50)	**0.50 (0.49**–**0.50)**
*Civil status*								
Married	0.63 (0.63–0.64)	0.60 (0.59–0.62)	0.52 (0.52–0.53)	**0.57 (0.57**–**0.58)**	0.62 (0.62–0.64)	0.60 (0.58–0.62)	0.53 (0.52–0.54)	**0.56 (0.56**–**0.57)**
Unmarried	0.16 (0.16–0.17)	0.19 (0.18–0.20)	0.23 (0.23–0.24)	**0.20 (0.20**–**0.20)**	0.18 (0.17–0.18)	0.19 (0.18–0.21)	0.24 (0.23–0.24)	**0.22 (0.21**–**0.22)**
Divorced	0.18 (0.17–0.18)	0.18 (0.17–0.19)	0.21 (0.21–0.22)	**0.20 (0.19**–**0.20)**	0.17 (0.17–0.18)	0.18 (0.17–0.20)	0.21 (0.20–0.21)	**0.20 (0.19**–**0.20)**
Widowed	0.023 (0.021–0.025)	0.025 (0.021–0.029)	0.028 (0.026–0.030)	**0.026 (0.025**–**0.027)**	0.022 (0.019–0.025)	0.025 (0.019–0.032)	0.025 (0.023–0.027)	**0.024 (0.023**–**0.026)**
*Education*								
Primary	0.15 (0.15–0.16)	0.17 (0.16–0.18)	0.22 (0.22–0.23)	**0.19 (0.19**–**0.19)**	0.15 (0.14–0.15)	0.15 (0.13–0.16)	0.20 (0.20–0.21)	**0.19 (0.18**–**0.19)**
Secondary	0.47 (0.46–0.48)	0.48 (0.47–0.49)	0.50 (0.49–0.50)	**0.48 (0.48**–**0.49)**	0.47 (0.46–0.48)	0.47 (0.45–0.49)	0.50 (0.49–0.51)	**0.49 (0.48**–**0.49)**
Tertiary	0.38 (0.37–0.38)	0.35 (0.34–0.36)	0.27 (0.27–0.28)	**0.32 (0.32**–**0.32)**	0.38 (0.37–0.39)	0.38 (0.36–0.40)	0.30 (0.29–0.30)	**0.33 (0.32**–**0.33)**
*Income*								
Q1	0.079 (0.076–0.083)	0.095 (0.087–0.10)	0.14 (0.14–0.15)	**0.11 (0.11**–**0.12)**	0.064 (0.059–0.070)	0.080 (0.0696–0.091)	0.14 (0.13–0.14)	**0.11 (0.11**–**0.12)**
Q2	0.11 (0.11–0.12)	0.13 (0.12–0.14)	0.16 (0.15–0.16)	**0.14 (0.14**–**0.14)**	0.11 (0.10–0.12)	0.11 (0.10–0.13)	0.15 (0.14–0.15)	**0.14 (0.13**–**0.14)**
Q3	0.25 (0.25–0.26)	0.26 (0.25–0.27)	0.28 (0.27–0.28)	**0.27 (0.26**–**0.27)**	0.26 (0.25–0.27)	0.25 (0.23–0.26)	0.27 (0.27–0.28)	**0.27 (0.26**–**0.27)**
Q4	0.55 (0.55–0.56)	0.52 (0.50–0.53)	0.42 (0.42–0.43)	**0.48 (0.48**–**0.49)**	0.57 (0.56–0.58)	0.56 (0.54–0.58)	0.44 (0.43–0.45)	**0.48 (0.48**–**0.49)**
*Origin*								
Immigrant	0.11 (0.11–0.11)	0.12 (0.12–0.13)	0.12 (0.12–0.13)	**0.12 (0.12**–**0.12)**	0.093 (0.087–0.10)	0.12 (0.11–0.13)	0.13 (0.13–0.13)	**0.12 (0.12**–**0.12)**
*Morbidity*								
FIT positive	0.12 (0.12–0.13)	0.14 (0.13–0.15)	–	**–**	–	–	–	**–**

Sociodemographic variables refer to the year before receiving the initial invitation and were obtained from Statistics Sweden. FIT positive refers to a hemoglobin concentration of at least 10 μg/g of feces in one of the submitted kits.

### 3.5 Substitution and extrapolation in SCREESCO

Compared to the unadjusted participant groups in SCREESCO, substitution somewhat improved the estimates of the population distributions of civil status, education, and income (Table 4). Improvements were also noticed for sex (in SCREESCO-FIT) and immigrant status.

**Table 4:** Proportions (95% confidence intervals) of different characteristics across invited and participants, and predicted values for the invited population based on early and late participants in SCREESCO.

	**Home test arm (SCREESCO-FIT)**				**Colonoscopy arm (SCREESCO-COL)**			
Variable	**True values in full invited population (n = 60,100)**	**Values among participants (n = 28,822)**	**Substitution** **based on late participants**	**Linear extrapolation**	**True values in full invited population (n = 30,395)**	**Values among participants (n = 10,541)**	**Substitution based on late participants**	**Linear extrapolation**
*Sex*								
Male	**0.50 (0.49**–**0.50)**	0.46 (0.45–0.46)	0.49 (0.48–0.51)	0.56 (0.54–0.57)	**0.50 (0.49**–**0.50)**	0.52 (0.51–0.53)	0.54 (0.52–0.56)	0.61 (0.57–0.65)
*Civil status*								
Married	**0.57 (0.57**–**0.58)**	0.63 (0.62–0.63)	0.62 (0.60–0.63)	0.60 (0.58–0.61)	**0.56 (0.56**–**0.57)**	0.62 (0.61–0.63)	0.61 (0.59–0.63)	0.57 (0.53–0.61)
Unmarried	**0.20 (0.20**–**0.20)**	0.17 (0.16–0.17)	0.18 (0.17–0.19)	0.20 (0.18–0.21)	**0.22 (0.21**–**0.22)**	0.18 (0.18–0.19)	0.19 (0.18–0.20)	0.22 (0.18–0.25)
Divorced	**0.20 (0.19**–**0.20)**	0.18 (0.17–0.18)	0.18 (0.17–0.19)	0.18 (0.17–0.19)	**0.20 (0.19**–**0.20)**	0.18 (0.17–0.18)	0.18 (0.17–0.19)	0.19 (0.16–0.22)
Widowed	**0.026 (0.025**–**0.027)**	0.023 (0.022–0.025)	0.024 (0.022–0.027)	0.025 (0.020–0.031)	**0.024 (0.023**–**0.026)**	0.023 (0.020–0.025)	0.024 (0.020–0.029)	0.029 (0.16–0.43)
*Education*								
Primary	**0.19 (0.19**–**0.19)**	0.16 (0.15–0.16)	0.16 (0.16–0.17)	0.18 (0.16–0.19)	**0.19 (0.18**–**0.19)**	0.15 (0.14–0.15)	0.15 (0.14–0.16)	0.15 (0.12–0.18)
Secondary	**0.48 (0.48**–**0.49)**	0.47 (0.47–0.48)	0.48 (0.47–0.49)	0.48 (0.47–0.50)	**0.49 (0.48**–**0.49)**	0.47 (0.46–0.49)	0.47 (0.45–0.49)	0.47 (0.53–0.51)
Tertiary	**0.32 (0.32**–**0.32)**	0.37 (0.36–0.38)	0.36 (0.35–0.37)	0.34 (0.33–0.36)	**0.33 (0.32**–**0.33)**	0.38 (0.37–0.39)	0.38 (0.36–0.40)	0.38 (0.34–0.42)
*Income*								
Q1	**0.11 (0.11**–**0.12)**	0.082 (0.079–0.086)	0.088 (0.083–0.093)	0.10 (0.09–0.11)	**0.11 (0.11**–**0.12)**	0.068 (0.063–0.073)	0.076 (0.068–0.084)	0.10 (0.075–0.12)
Q2	**0.14 (0.14**–**0.14)**	0.12 (0.11–0.12)	0.13 (0.12–0.13)	0.14 (0.13–0.15)	**0.14 (0.13**–**0.14)**	0.11 (0.10–0.12)	0.11 (0.10–0.12)	0.12 (0.095–0.15)
Q3	**0.27 (0.26**–**0.27)**	0.26 (0.25–0.26)	0.26 (0.25–0.27)	0.26 (0.25–0.27)	**0.27 (0.26**–**0.27)**	0.25 (0.25–0.26)	0.25 (0.23–0.26)	0.24 (0.20–0.27)
Q4	**0.48 (0.48**–**0.49)**	0.55 (0.54–0.55)	0.53 (0.52–0.54)	0.51 (0.49–0.52)	**0.48 (0.48**–**0.49)**	0.57 (0.56–0.58)	0.56 (0.54–0.59)	0.55 (0.51–0.59)
*Origin*								
Immigrant	**0.12 (0.12**–**0.12)**	0.11 (0.11–0.12)	0.12 (0.11–0.12)	0.13 (0.12–0.14)	**0.12 (0.12**–**0.12)**	0.10 (0.094–0.11)	0.11 (0.10–0.12)	0.15 (0.12–0.18)
*Morbidity*								
FIT positive	**–**	0.13 (0.12–0.13)	0.13 (0.13–0.14)	0.14 (0.13–0.15)	**–**	–	–	–

Sociodemographic variables refer to the year before receiving the initial invitation and were obtained from Statistics Sweden. FIT positive refers to a hemoglobin concentration of at least 10 μg/g of feces in one of the submitted kits.

Compared to the baseline of unadjusted participants, extrapolation produced less accurate estimates of the population distributions of sex, especially in SCREESCO-COL, where the share of males was substantially overestimated. The extrapolation-based estimates of the distributions of civil status were rather accurate, however, whereas those for education and income were rather accurate in the FIT arm but less so in the COL arm. Results for immigrant status were rather accurate in the FIT arm but overestimated the share of immigrants in the COL arm. Neither substitution nor extrapolation made much difference for the estimated probability of a positive FIT result, as compared to the baseline.

## 4 Discussion

In this article, we investigated the applicability of the continuum of resistance model in two large Swedish screening studies: STROKESTOP II and SCREESCO. With the goal of reproducing the distributions of various characteristics in the full invited populations without utilizing information on non-participants, we applied both a substitution and an extrapolation-based method. Primarily considering background characteristics observed in population registers, we compared our results with the true values in the full invited populations. In turn, we provided clues as to whether the models may also be able to reproduce population distributions of outcomes not observed in the full populations, such as risk factors that were screened for, or, in the longer term, benefits of the screenings. Our investigation is one of the first on the continuum of resistance model in the context of screenings or health examinations, a context that differs from surveys as the decision to participate may be influenced by different factors (McLachlan et al., 2012, McQueen et al., 2010).

Overall, we found mixed support for the continuum of resistance model. In STROKESTOP II, the substitution and extrapolation methods allowed us to come closer to the true population distributions of immigrant status, the Charlson comorbidity index, and with the extrapolation method also education and income. Nevertheless, discrepancies remained, not least for the Charlson comorbidity index, a variable that may be strongly related to long-term health outcomes and the benefits of screening (Charlson et al., 1987, Marventano et al., 2014, Tessier et al., 2008). In SCREESCO-FIT, the methods brought us closer to the population distributions of civil status, education, income, and immigrant status; however, fewer improvements were seen in SCREESCO-COL. Applying our methods to outcomes that were screened for in STROKESTOP II or SCREESCO did not generally produce estimates much different from those observed among the participants, but it is unknowable whether this reflects a high degree of representativeness with respect to these outcomes, or if the continuum of resistance model is less applicable to these.

The main goal of STROKESTOP II and SCREESCO is to examine the effects of screening programs on long-term outcomes such as mortality and incidence of ischemic stroke and colorectal cancer (Engdahl et al., 2017, Forsberg et al., 2022). In their analyses of these outcomes, investigators will compare the full invited populations to control groups of uninvited, estimating intention-to-treat (ITT) effects, that is, average effects of being assigned to screening. The ITT is useful because it is not subject to confounding, but since not everyone who was invited to screening agreed to participate, it is not the same as the average effect of the actual screening. The latter quantity is more difficult to determine, and a simple comparison of participants and non-participants would likely be subject to confounding bias (Hernán and Hernández-Díaz, 2012). The methods we have described could potentially be useful to determine the effects of actual screening, however. Applying substitution or extrapolation to a long-term outcome, an investigator could estimate the outcome distribution in the full invited population, given the counterfactual scenario where everyone in the invited population were screened. The average population effect of screening could then be estimated by contrasting the results from this counterfactual scenario with the corresponding long-term outcomes in the control group of individuals not invited to the screening. Future work on STROKESTOP II, SCREESCO, and other randomized, population-based screening studies may apply such a method to try to uncover the efficacy of screening, while keeping in mind that the method is far from guaranteed to be successful, as hinted by the somewhat limited success to reproduce the distributions of background characteristics in our study.

Statistical modelling can never fully replace collection of real observed data. Data collectors should consider various measures to maximize participation rates and to reduce the tendency of certain groups to participate to a lesser extent. In the context of screening this may be done, for example, by adding screening centers to disadvantaged neighborhoods (Gudmundsdottir et al., 2021) or increasing the awareness of screening and its benefits (Honein-AbouHaidar et al., 2016, Shokar et al., 2016). Besides this, record linking to sources with detailed sociodemographic or healthcare data can allow researchers to set recruitment goals (Bolen et al., 2006) or to reweight their samples with respect to observed predictors of participation (Gudmundsdottir et al., 2022). An interesting avenue for future work is to combine the continuum of resistance model with reweighting, potentially improving representativity even further.

## 5 Consent to participate

Participants in both STROKESTOP II and SCREESCO were provided information on how their data would be used and signed informed consent documents. Anonymized data on the invited populations were collected from official registries.

## Funding

This work was supported by Swedish Research Council for Health, Working Life and Welfare (FORTE; grant 2020–00962 to Carl Bonander) and the Swedish Cancer Society (grant 20 0719 to Ulf Strömberg). The funders were not involved in the study concept and design, data collection and analysis, interpretation of results, drafting of the manuscript, or the decision to submit the manuscript for publication. The content of the paper is solely the responsibility of the authors and does not necessarily represent the views of the funding agencies.

## CRediT authorship contribution statement

**Anton Nilsson:** Conceptualization, Formal analysis, Investigation, Methodology, Writing - original draft, Writing - review & editing. **Ulf Strömberg:** Funding acquisition, Methodology, Writing - review & editing. **Jonas Björk:** Methodology, Writing - review & editing. **Anna Forsberg:** Resources, Writing - review & editing. **Kaisa Fritzell:** Resources, Writing - review & editing. **Katrin Kemp Gudmundsdottir:** Resources, Writing - review & editing. **Johan Engström:** Resources, Writing - review & editing. **Carl Bonander:** Funding acquisition, Data curation, Methodology , Writing - review & editing.

## Declaration of Competing Interest

The authors declare the following financial interests/personal relationships which may be considered as potential competing interests: Johan Engdahl has received speaker/consultancy fees from Pfizer, Roche Diagnostics, Philips, and Piotrode. Katrin Kemp Gudmundsdottir has received speaker/lecture fees from Pfizer, Boehringer Ingelheim, and Roche Diagnostics. Anton Nilsson, Ulf Strömberg, Jonas Björk, Anna Forsberg, Kaisa Fritzell, and Carl Bonander report no conflicts of interest.

## Data Availability

Code will be shared by the corresponding author upon request. Researchers with an ethical approval can also contact the corresponding author to gain access to the data.
